# Using organ-on-a-chip technology to study haemorrhagic activities of snake venoms on endothelial tubules

**DOI:** 10.1038/s41598-024-60282-5

**Published:** 2024-06-04

**Authors:** Mátyás A. Bittenbinder, Flavio Bonanini, Dorota Kurek, Paul Vulto, Jeroen Kool, Freek J. Vonk

**Affiliations:** 1 Naturalis Biodiversity Center, 2333 CR Leiden, The Netherlands; 2 AIMMS, Division of BioAnalytical Chemistry, Department of Chemistry and Pharmaceutical Sciences, Faculty of Sciences, Vrije Universiteit Amsterdam, De Boelelaan 1085, 1081HV Amsterdam, The Netherlands; 3 Mimetas, Leiden, The Netherlands

**Keywords:** Snakebite, Envenoming, Tissue-damaging activities, Haemorrhage, Organ-on-chip, 3D cell culture, Microfluidics, Cell death, Cellular imaging

## Abstract

Snakebite envenomation is a major public health issue which causes severe morbidity and mortality, affecting millions of people annually. Of a diverse range of clinical manifestations, local and systemic haemorrhage are of particular relevance, as this may result in ischemia, organ failure and even cardiovascular shock. Thus far, in vitro studies have failed to recapitulate the haemorrhagic effects observed in vivo. Here, we present an organ-on-a-chip approach to investigate the effects of four different snake venoms on a perfused microfluidic blood vessel model. We assess the effect of the venoms of four snake species on epithelial barrier function, cell viability, and contraction/delamination. Our findings reveal two different mechanisms by which the microvasculature is being affected, either by disruption of the endothelial cell membrane or by delamination of the endothelial cell monolayer from its matrix. The use of our blood vessel model may shed light on the key mechanisms by which tissue-damaging venoms exert their effects on the capillary vessels, which could be helpful for the development of effective treatments against snakebites.

## Introduction

Snakebite is one of the leading global health crises known to date, which claim between 81,000 and 138,000 lives annually, particularly in the (sub)tropical regions of this world^1,2^. Snake venom is comprised of a complex mixture of proteins and peptides, which poses a substantial threat to human life, causing significant morbidity and mortality. Snakebite morbidity, which is mainly caused by the toxins that directly or indirectly destroy cells, may cause permanent disability, which includes severe tissue loss (i.e., muscle and skin), chronic renal diseases and blindness^1,3–6^. Morbidity is estimated to occur in at least 400,000 snake bite victims annually^1,2,5,7^.

Many venoms have tissue-damaging activities that may result in a range of pathologies, including muscle and skin necrosis, acute kidney injury and haemorrhage^1,7–9^. The most medically relevant pathology caused by the tissue-damaging components in snake venoms is local and systemic bleeding^1,10^. Local and systemic haemorrhage may further promote ischemia, which may cause acute kidney injury and organ failure and could even lead to cardiovascular shock^1,11,12^. Haemorrhage is a rapid event in vivo with capillary endothelial cells showing drastic structural alterations within minutes^13,14^.

However, the same tissue-damaging components do not necessarily induce rapid toxicity to endothelial cells in vitro. The discrepancy between these two observations may arise from the fact that traditional in vitro systems do not take into account the mechanical action of haemodynamic forces acting on the endothelial cells, as is the case with in vivo systems^12,15,16^. To date, the study of tissue-damaging effects of snake venom toxins has been primarily based on two-dimensional cell culture models. These do not have the tubular morphology of vasculature found in vivo and lack important environmental cues from the cellular microenvironment, such as interaction with an extracellular matrix (ECM) and exposure to flow^17,18^. In an attempt to address this discrepancy, innovative approaches are required that advance our understanding of venom-induced haemorrhage and can be utilised to develop effective treatments. The development of snakebite treatments focusing on the neutralisation of the haemorrhagic effects of snake venom components relies on robust and quantitative in vitro models of blood vessels.

Organ-on-chip is an emerging technology that utilises microengineering to create three-dimensional models in microfluidic channel networks. In contrast to cells grown in 2D, blood vessels grown in microfluidic channels enable the inclusion of several important physiological parameters during cell culture, such as 3D tubular morphology, fluid perfusion, inclusion of ECM and exposure to biochemical gradients^19^. Organ-on-a-chip assays have been successfully used for the formation of perfused vascular models, including angiogenesis^20,21^, T-cell migration^22^, monocyte adhesion^23^, vascularisation of spheroids^24^ and real-time methods to study vascular barrier function^25^. In this latter study, organ-on-chip is combined with high-content imaging systems that enable direct monitoring of vascular barrier, viability and morphology.

In this study, we aimed to use a human blood vessel model grown in an organ-on-chip platform to simulate the hemorrhagic effect of different snake venoms while assessing their direct toxicity to endothelial cells. For this, we exposed microfluidic human blood vessels to a panel of four venoms from four different snake species. We used fluorescent tracer molecules and image analysis to assess endothelial barrier function and monitor vascular leakage following venom exposure. Furthermore, we assessed cellular viability and vessel morphology to evaluate the tissue-damaging activities of the snake venoms comprehensively. This allowed us to differentiate between different mechanisms of action by which venoms cause blood vessel disruption.

We found two distinct mechanisms by which the blood vessel is being affected: delamination of the endothelial cell monolayer from its matrix and disruption of the endothelial cell membrane. The workflow presented here could be utilised in the routine assessment of snake toxicity and its tissue-damaging and proteolytic effects and will be helpful for the development of effective snakebite treatments.

## Materials and methods

### Venoms

Venoms were sourced from the library of the Faculty of Science, BioAnalytical Chemistry, Vrije Universiteit Amsterdam (VU). This library contains samples obtained and subsequently provided by the Liverpool School of Tropical Medicine (LSTM), National University of Singapore (NUS) and captive breeders. The snake venoms used in this study came from the following viper (Viperidae) and elapid (Elapidae) species: *Echis ocellatus* (West African carpet viper, Nigeria), *Bungarus multicinctus* (many-banded krait, locality unknown), *Naja mossambica* (Mozambique spitting cobra, captive bred) and *Naja naja* (Indian cobra, captive bred). Venoms from VU and NUS were lyophilised immediately after milking, then freeze-dried and stored at − 80 °C. LSTM venoms were extracted, stored overnight at − 20 °C, then lyophilised and stored at 4 °C for the long term. Samples were reconstituted in milli-Q (mQ) H_2_O to the desired stock solutions, depending on the type of assay. These solutions were then aliquoted and subsequently snap-frozen in liquid nitrogen and stored at − 80 °C until use. All venoms were sourced in accordance with the Nagoya protocol, where applicable^26^.

### OrganoPlate culture

OrganoReady Blood Vessel HUVEC 3-lane 64 plates (Mimetas B.V, MI-OR-BV-02) were cultured according to the manufacturer’s instructions. The medium was replaced on the day of receiving to OrganoMedium HUVEC-BM (Mimetas B.V.) on the day of receiving the plates. OrganoReady Blood Vessel HUVEC 3-lane 64 are ready-to-use HUVEC tubes in OrganoPlates that follow a similar process for ECM seeding (rat tail collagen 1 at 4 mg/mL), endothelial cell seeding and establishment of perfusion through the tubules as described by Duinen et al. for the OrganoPlate 3-lane 40^19^. The perfusion flow was maintained by placing the plate on an OrganoFlow rocker (Mimetas B.V., MI-OFPR-L) set at 14 degrees with 8-min intervals optimised for the 3-lane 64 in a humidified incubator (37 °C, 5% CO_2_). This allows the perfusion of the medium through the endothelial tube in an intermittent, bi-directional flow profile with peaks of shear stress of approximately 0.17 Pa. Plates were kept in a humidified incubator (37 °C, 5% CO_2_) for 5 days prior to venom exposure with a complete medium change (50 µL in each in-and outlets) every 2–3 days.

### Vascular leakage assessment

**Figure 1:**
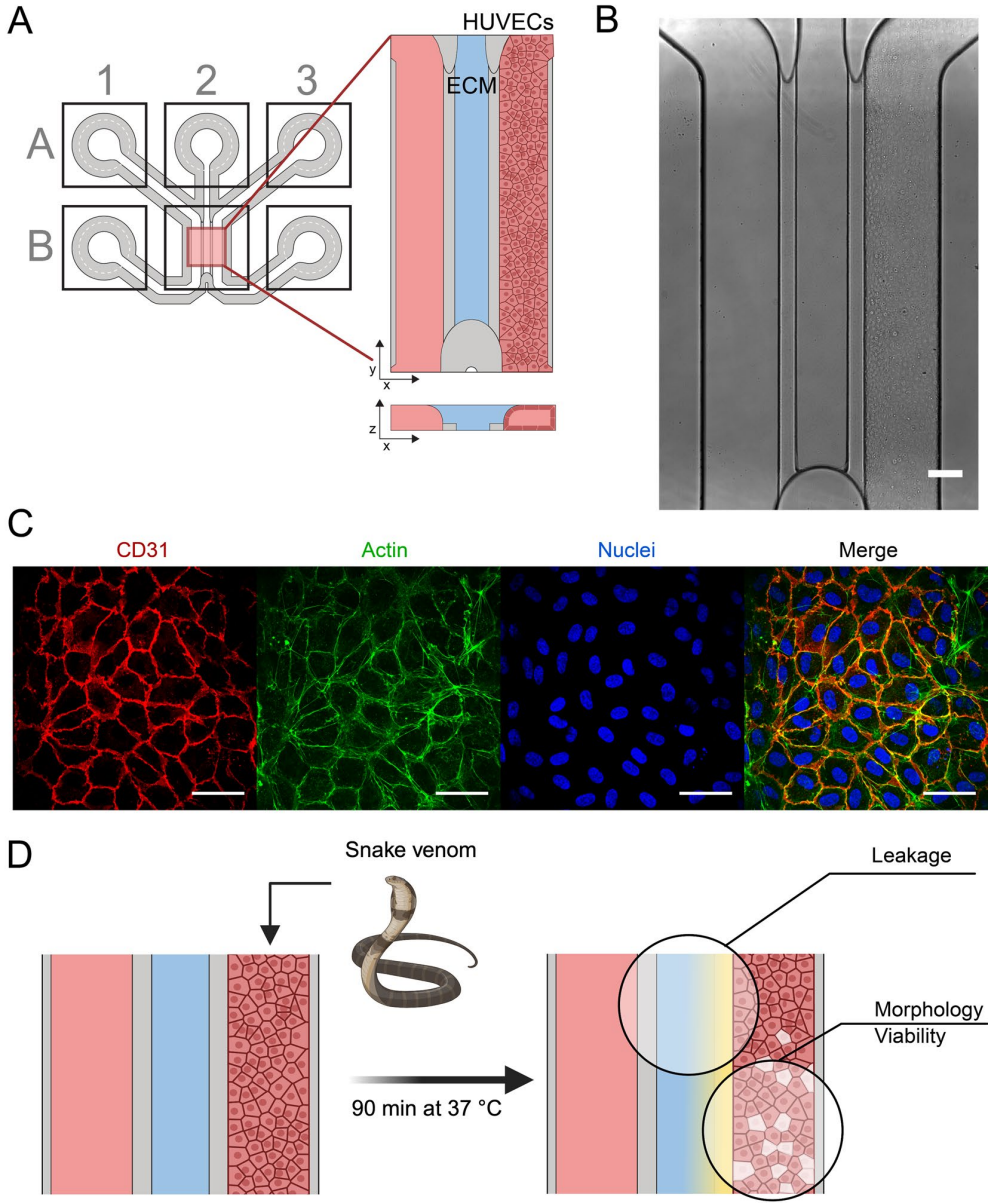
Set-up for studying haemorrhagic activities in the OrganoPlate 3-lane 64. (**A**) Schematic of the channel arrangement of one culture chip within the OrganoPlate 3-lane 64. (**B**) Brightfield image of a HUVEC blood vessel grown against the collagen-I-filled middle channel. The scale bar represents 200 μm. (**C**) Immunofluorescent microscopy images showing the expression of platelet cell adhesion molecule CD31, actin and nuclei of the grown blood vessel. The scale bar represents 50 μm. (**D**) Schematic overview of the experimental setup. Snake venom is added in combination with fluorescent tracers and live imaging dyes. Live microscopy is used to assess endothelial leakage, morphology and viability.

A mixture of 10 μg/mL, 1 μg/mL snake venom or 1:100 mQ water (as vehicle control) was prepared in OrganoMedium HUVEC-BM with 0.25 mg/mL TRITC-labelled 150 kDa Dextran (Sigma, T1287). The medium was then aspirated from all wells of the OrganoPlate, and 20 μL HBSS (Thermo, 14025092) was added to the left channel wells (see A1 and B1 in Fig. 1A). 20 μL of venom or control solution was added to the bottom well of the right channel (B3 in Fig. 1A), followed by 40 μl in the top well of the right channel (see A3 in Fig. 1A) in a total of 4 different chips/condition. The plate was then quickly introduced in an ImageXpress Micro XLS microscope (Molecular Devices) with temperature control set at 37 °C. Fluorescent images were acquired every 2 min for 30 min. The plate was then placed back in a humidified incubator (37 °C, 5% CO_2_) on the OrganoFlow rocker. Additional single timepoint images were acquired 60 min and 90 min after venom addition. Fluorescence intensity was quantified as average intensity from the gel channel adjacent to the endothelial tubule using Fiji^27^.

### Assessment of endothelial tubule morphology

A staining solution was prepared in OrganoMedium HUVEC-BM with 2 drops/mL Propidium Iodide Ready Flow™ Reagent (Thermo, R37169), 1:500 SPY650-FastAct™ (reconstituted following manufacturer’s instructions), 1:500 Calcein-AM (Thermo, C3099) and 1:1000 Hoechst (Thermo, H3570). Medium from all wells was removed and replaced with 50 μL of staining solution and incubated in an incubator for 1 h prior to snake venom addition. Bright-field and fluorescent images were acquired using an ImageXpress Micro XLS microscope with a 10X objective. Then, a mixture of 10 μg/mL, 1 μg/mL snake venom or 1:100 Milli-Q water (as vehicle control) was prepared in a staining solution. The staining solution was aspirated from the left channel wells of the OrganoPlate (see A1 and B1 in Fig. 1A) 20 μl of venom or control solution was then added to the bottom well of the right channel (see B3 in Fig. 1A), followed by 40 μl in the top well of the right channel (see A3 in Fig. 1A) in a total of 3 different chips/condition. The plate was placed back on the rocker in the incubator, and images were again acquired after 90 min of incubation. For time-lapse acquisition, venom was diluted to 100 μg/mL in a staining solution, and single-slice images were acquired every 10 s for 300 s using a Micro XLS-C confocal microscope (Molecular Devices) set at 37 °C.

### Viability quantification

The propidium iodide (PI) and Hoechst signals were thresholded to a black-and-white image using Fiji. Watershed was applied to segment overlapping nuclei, which were counted in each channel using the built-in analyse particle function. Viability was then expressed as a percentage of the total number of nuclei minus PI-positive nuclei.

### Statistical analysis

Significant differences between control and experimental groups without multiple factors were assessed using 1-way ANOVA. Statistical analysis was performed using GraphPad Prism 6.

## Results

### Assessment of barrier integrity (i.e., venom-induced vascular leakage)

Figure 1 shows the setup used to assess haemorrhagic effects of snake venoms. A blood vessel was grown in a microfluidic channel by seeding HUVECs against an extracellular matrix, forming a perfused lumenised tubule after 5 days of culture (see Fig. 1A,B). The resulting blood vessel consists of a dense endothelial layer with extensive cell junctions between adjacent cells, as indicated by the expression of CD31 (see Fig. 1C). The lumen of the vessel structure was exposed to snake venom, a fluorescent tracer molecule and live imaging dyes for toxicity and morphological assessment (see Fig. 1D). Barrier function of the blood vessel was assessed by observing leakage of the fluorescent tracer (FITC-dextran, 150 kDa) to the lumen and measuring leakage into the adjacent gel channel (Supplementary Fig. 1).

**Figure 2:**
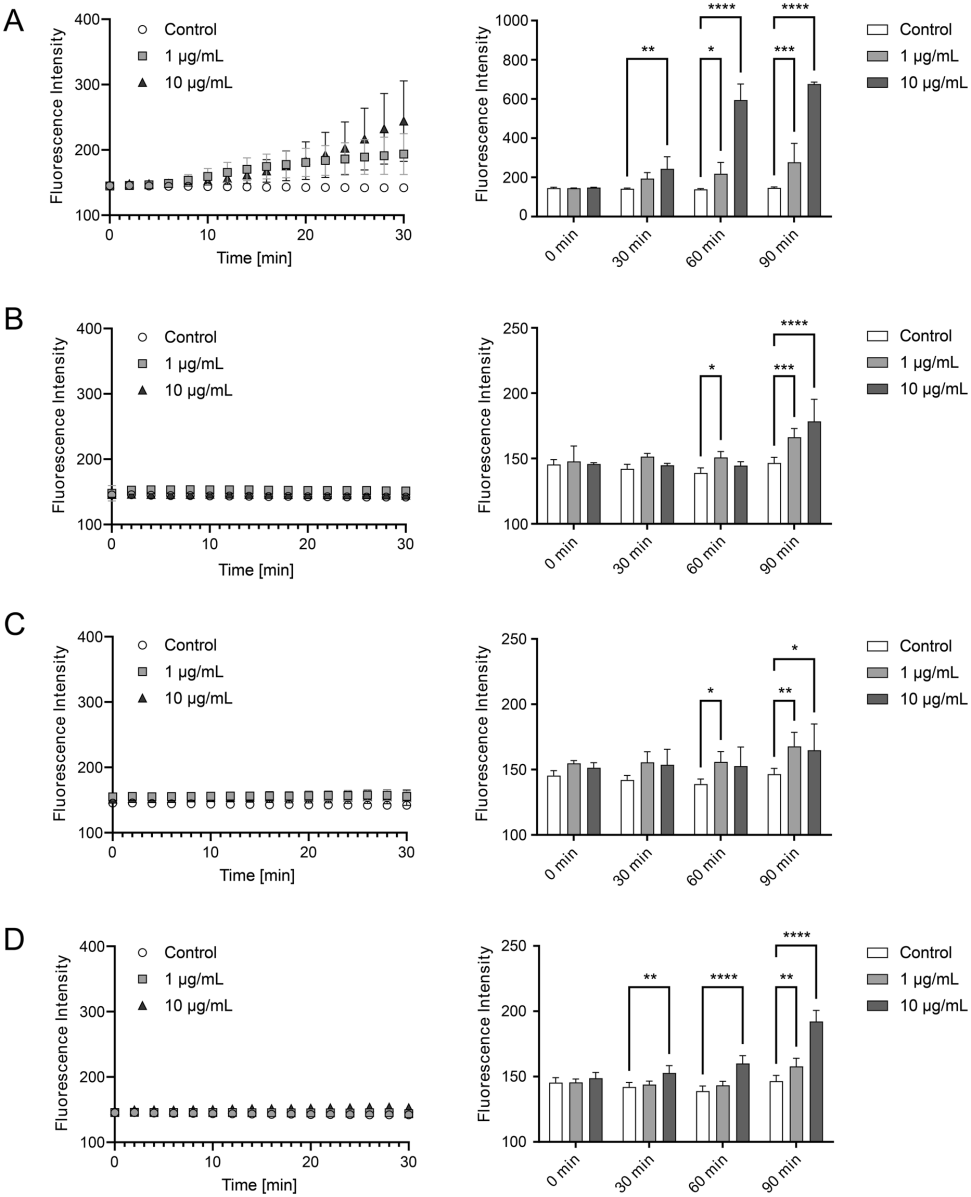
Assessment of venom-induced vascular leakage. Fluorescence intensity was quantified as the average intensity measured from the middle ECM channel as an indication of vascular leakage. The left panel shows the time course of vascular leakage for the first 30 min after venom addition, and the right panel shows fluorescence intensity at 0, 30, 60 and 90 min after venom addition of (**A**) *E. ocellatus,* (**B**) *N. mossambica,* (**C**) *B. multicinctus,* (**D**) *N. naja*. Measurements are presented as the mean of three individual experiments (N=4); mean ± SD; *p < 0.05, **p < 0.01, ***p < 0.001, ****p < 0.001, 1-way ANOVA, venom exposed vs control).

Figure 2 shows the impact on blood vessel barrier function from a panel of snake venoms. Snake venom of *E. ocellatus* induced extensive leakage within minutes in both 1 and 10 μg/mL concentrations (see Fig. 2A, left). Tubule leakage continued to increase at 60 and 90 min after venom addition (see Fig. 2A, right). *N. mossambica* did not induce vascular leakage during the first 30 min (see Fig. 2B, left), but significant outflux of dextran was observed after 60 and, in particular, 90 min of exposure (see Fig. 2B, right). *B. multicinctus* caused significant leakage at 60 and 90 min of exposure, but not in a dose-dependent manner (see Fig. 2C). *N. naja* at 10 µg/mL induced significant leakage after 30 and 60 min, and at 90 min, both tested concentrations caused significant dextran leak (see Fig. 2D).

### Morphological assessment of venom exposure to endothelial tubules

**Figure 3.**
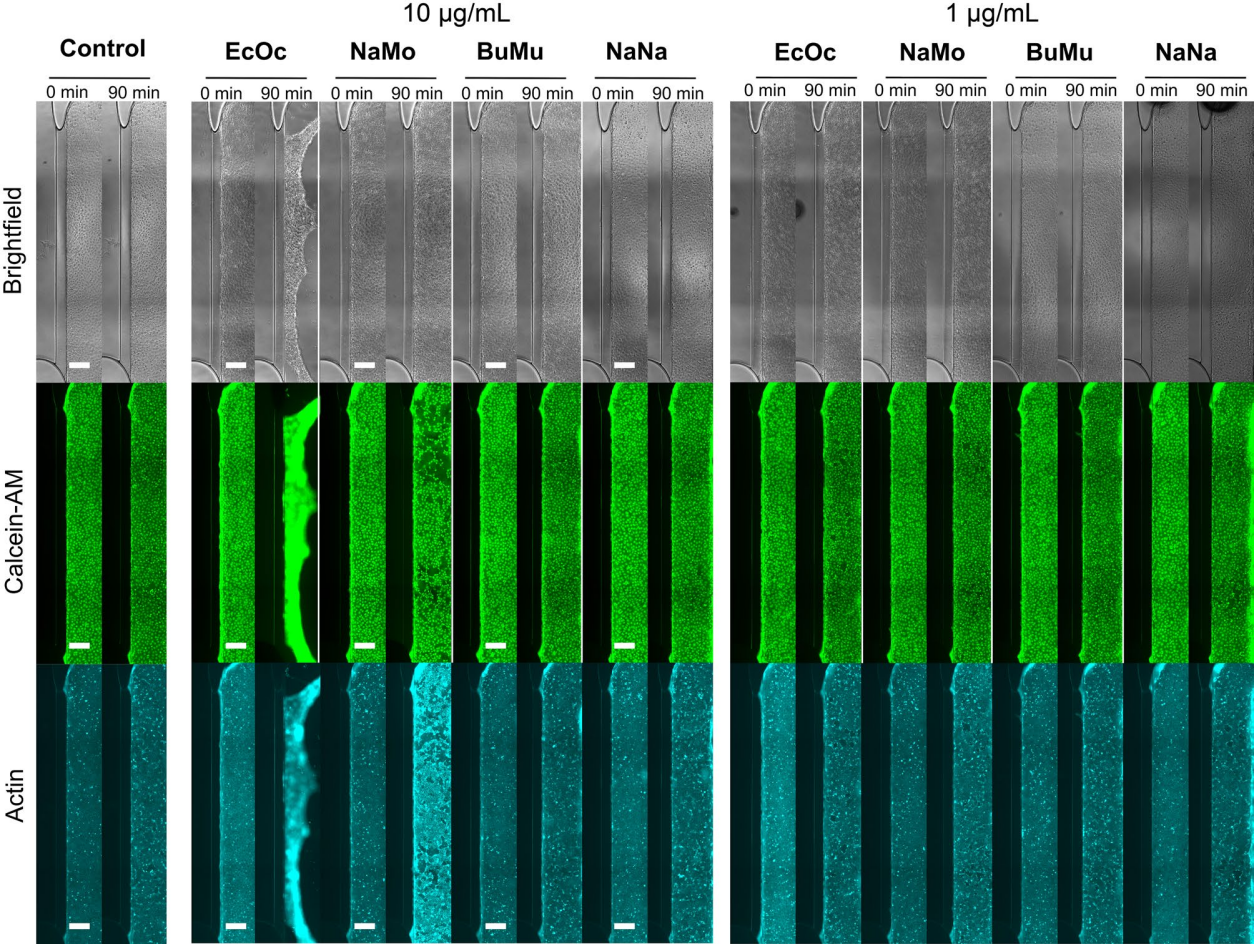
Morphological assessment of endothelial tubules after 90 min of snake venom exposure. Brightfield and immunofluorescent microscopy images show the morphology of the endothelial vessels at 0 min and 90 min after exposure to 1 and 10 µg/mL of snake venom, compared to control. The scale bar represents 200 μm. Abbreviations: EcOc, *E. ocellatus*; NaMo*, N. mossambica*; BuMu, *B. multicinctus*; NaNa, *N. naja*.

We assessed the effect of the venoms on the morphology and structure integrity of the endothelial tubes by microscopy. Endothelial tubes were incubated for 90 min with venoms in combination with Calcein-AM, PI and a live actin probe (see Fig. 3). We observed drastic structural disruptions for tubes exposed to 10 µg/mL *E. ocellatus* as indicated by delamination and collapse of the structure. In contrast, 1 µg/mL caused smaller, localised gaps in the endothelial layer. The other venoms did not seem to induce the same general structural damage to the endothelial tubules. Still, we observed marked vessel disruption in tubules exposed to 10 µg/mL, as indicated by an extensive loss of Calcein-AM and actin signal in parts of the cell layer. Notably, we observed an increase in actin signal in the remaining cells compared to the control.

### Assessment of direct cytotoxic activity

We then assessed whether venoms had a direct cytotoxic effect (i.e., toxins that have a toxic effect directly on the cells) or an indirect cytotoxic effect (e.g., by affecting the ECM, which could eventually lead to cell injury) on the endothelial cells.

**Figure 4:**
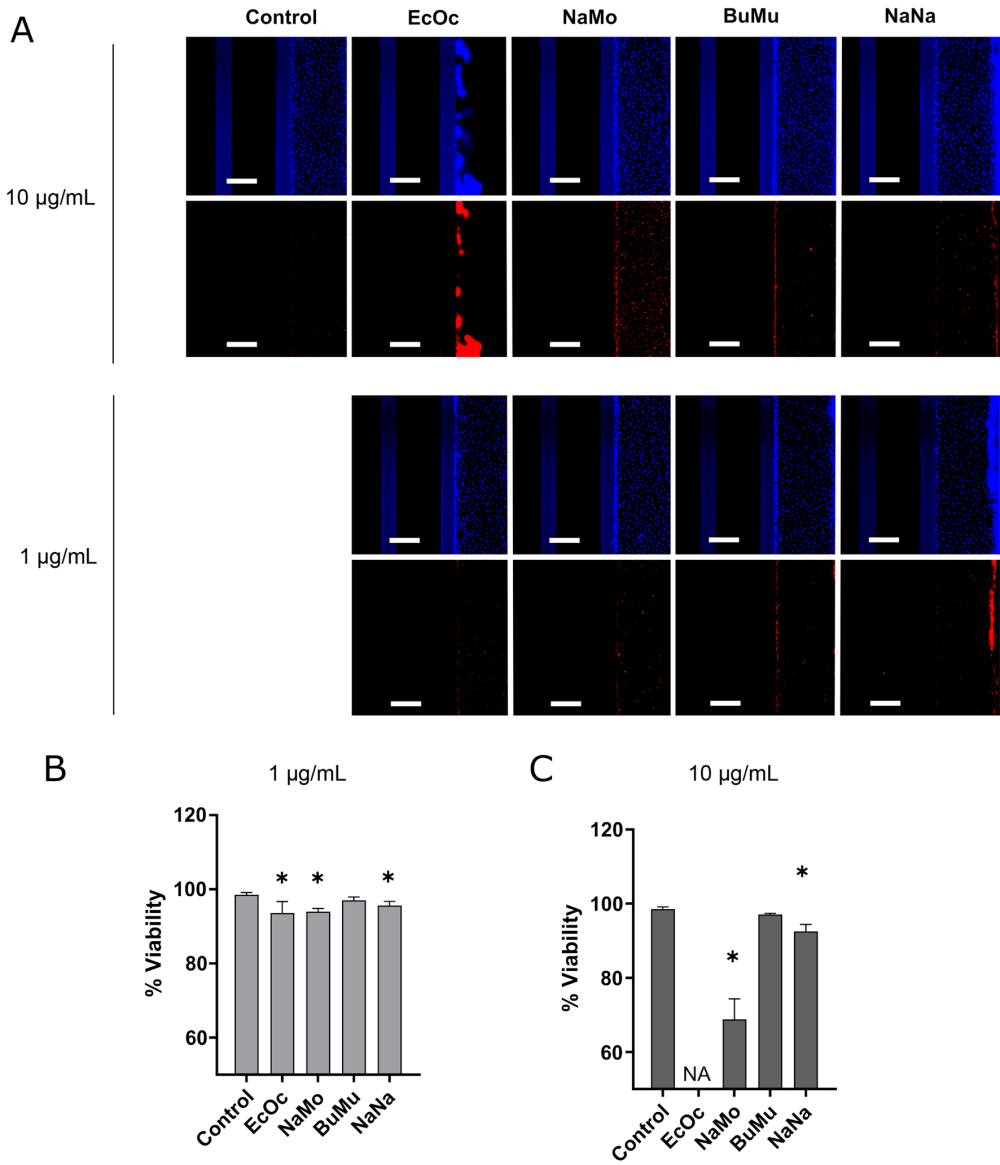
Viability assessment 90 min after snake venom exposure. (**A**) Immunofluorescent microscopy images showing the difference in morphology of the endothelial vessels 90 min after exposure to 1 µg/mL and 10 µg/mL of snake venom, compared to control. Hoechst staining is shown in blue (top panels) and PI in red (lower panels). The scale bar represents 200 μm. (B+C): Quantitative assessments of four species at 1 μg/mL, (**B**) and 10 μg/mL, (**C**) are shown as bar graphs. Measurements are presented as the mean of three Individual experiments (N = 4); mean ± SD; *p < 0.05, 1-way ANOVA, venom exposed vs. control). Abbreviations: EcOc, *E. ocellatus*; NaMo*, N. mossambica*; BuMu, *B. multicinctus*; NaNa, *N. naja*.

To distinguish direct cytotoxicity from indirect effects (matrix remodelling, contraction), we performed a viability assessment by staining and quantifying the number of total and dead cells using Hoechst and PI, respectively (see Fig. 4A). 90 min after 1 µg/mL venom exposure, we observed a slight but significant reduction in viability for *E. ocellatus* (93%), *N. mossambica* (94%) and *N. naja (*95%), but no change in viability was observed for *B. multicinctus* compared to control (see Fig. 4B). Similarly, 10 µg/mL caused a decrease in viability for *N. mossambica* (70%) and *N. naja* (92%). For exposure to *E. ocellatus,* viability could not be determined as tubule contraction prevented from quantifying individual nuclei (see Fig. 4C).

**Figure 5:**
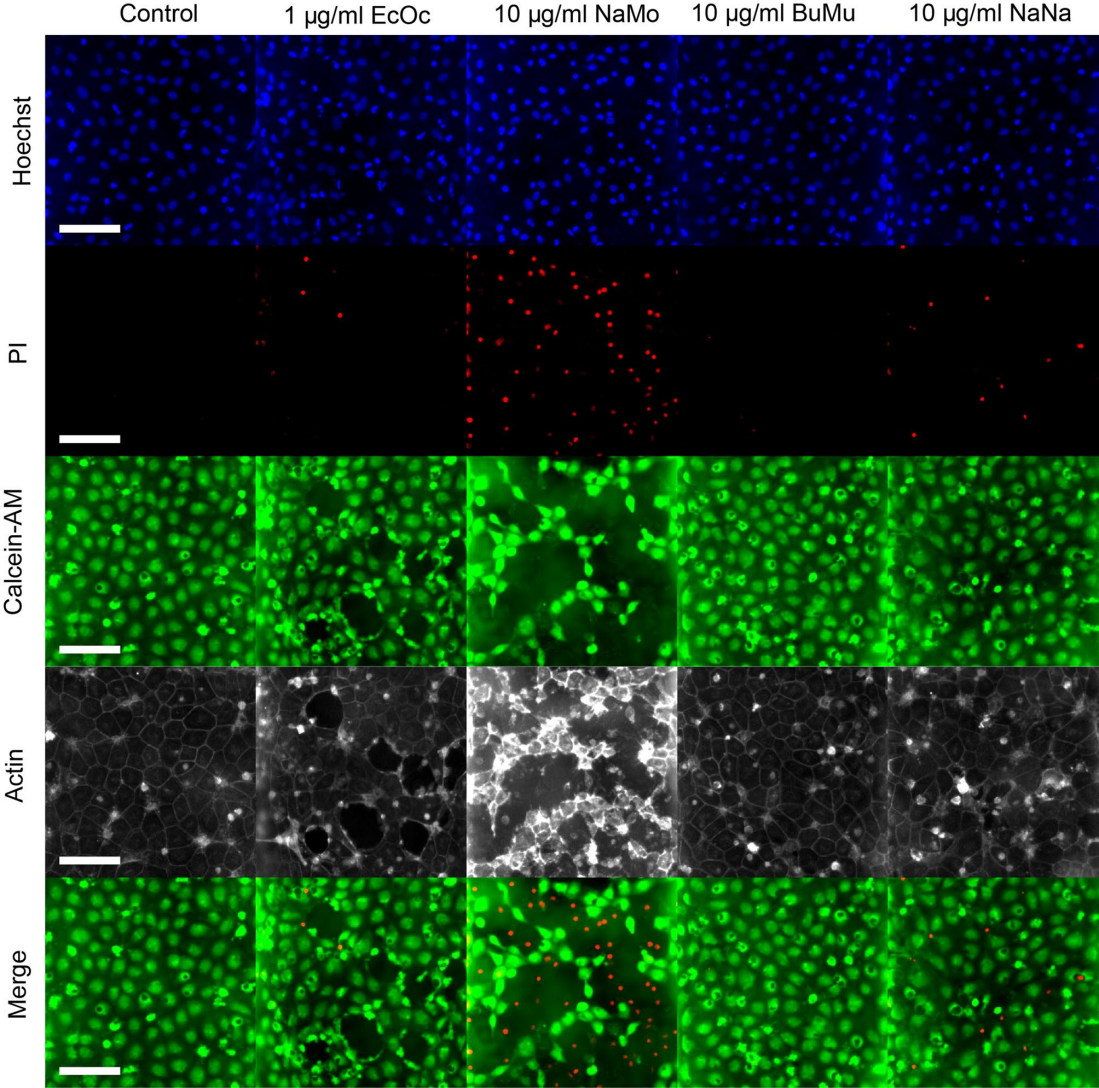
Morphological assessment of direct cytotoxic activity. Immunofluorescent microscopy images showing the difference in morphology of the endothelial vessels after 90 min of exposure to snake venom (1 μg/mL or 10 μg/mL), compared to control. Hoechst staining is shown in blue, PI in red, Calcein-AM in green and live-actin in white. The lower panels represent an image in which all four channels are merged. The scale bar represents 100 μm. Abbreviations: EcOc, *E. ocellatus*; NaMo, *N. mossambica*; BuMu, *B. multicinctus*; NaNa, *N. naja*.

At the cellular level, notable differences emerge between the toxic effects of the different venoms in terms of cell morphology. Although at 10 µg/mL, *E. ocellatus* caused complete collapse of the endothelial vessels, 1 µg/mL was sufficient to cause apparent gaps in the Calcein-AM and actin stain of the endothelial layer (see Fig. 5). Interestingly, dead cells were observed at the edges of these voids but not within them. Similarly, 10 µg/mL *N. mossambica* caused significant disruption of the cell layer, but, in contrast to *E. ocellatus*, dead cells were clearly visible inside these spaces. Although visibly less potent, we observed a similar pattern for *N. naja* while *B. multicinctus* did not seem to alter the cell layer’s morphology to any significant extent.

**Figure 6:**
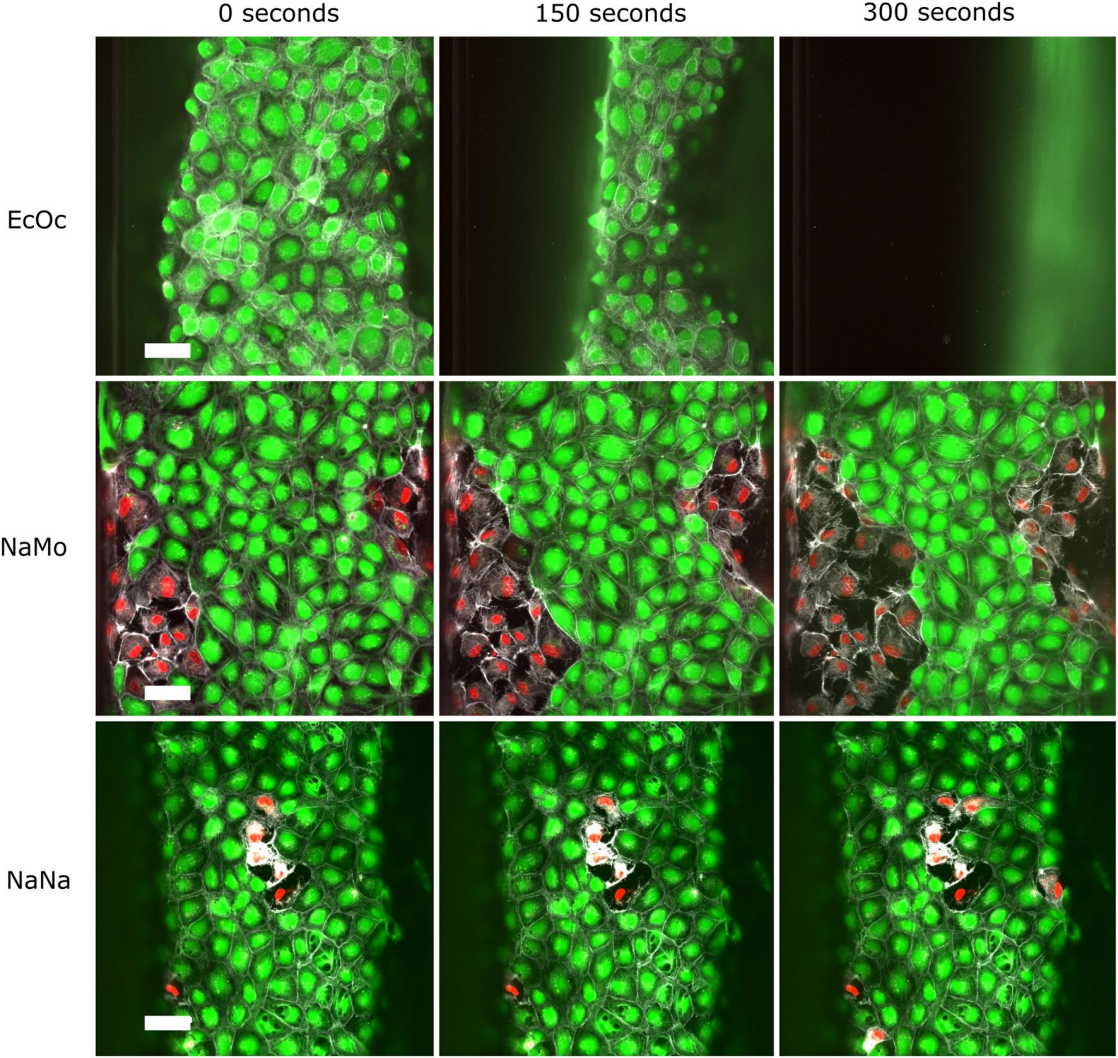
Timelapse of high venom dose exposure on endothelial tubules Immunofluorescent microscopy images show the difference in morphology of the endothelial vessels after 0, 150, and 300 s of exposure to 100 μg/mL of snake venom compared to the control. PI is shown in red, Calcein-AM is shown in green, and live-actin is shown in white. The scale bar represents 50 μm.

To further characterise and confirm the morphological changes at the cellular level as a result of venom exposure, we performed live imaging of endothelial tubules exposed to a higher concentration of 100 µg/mL of venom for 300 s. We observe that *E. ocellatus* delaminates the endothelial tubules without inducing significant cell death (see Fig. 6, Supplementary Video). In line with previous observations, *N. mossambica* caused direct cytotoxicity, as indicated by a simultaneous loss of Calcein-AM stain and emergence of PI signal in the nucleus. Notably, cell death seemed to occur preferentially in cells adjacent to already dead cells, which could be a distinct front of cell death propagation. *N. naja* exposure also led to direct cytotoxicity, although to a lesser magnitude compared to *N. mossambica*, and no clear cell death propagation was observed at this concentration.

## Discussion

**Figure 7:**
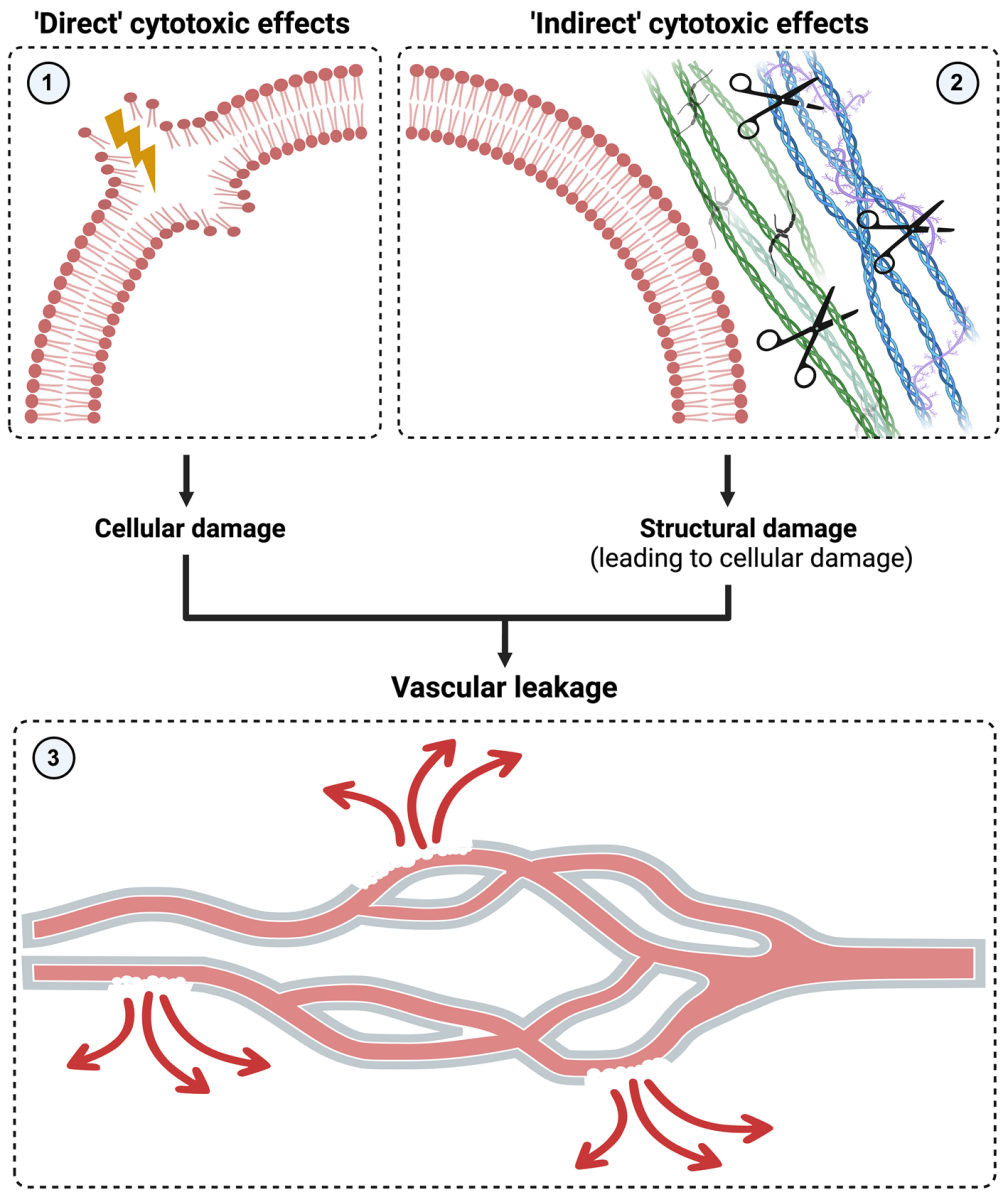
Schematic overview showing the mechanisms by which tissue-damaging toxins exert their effects. (1) ‘Direct’ cytotoxic effects cause cellular damage by acting directly on the endothelial cells (e.g., by affecting the membrane integrity). (2) The indirect cytotoxic effects (i.e., degradation of various ECM components) cause structural damage, which may contribute to cellular damage indirectly by affecting the stability of endothelial cells. (3) Both mechanisms may ultimately lead to vascular leakage. The image was created via www.biorender.com (with permission).

In this study, we utilised an “organ-on-a-chip” approach to study the effect of a panel of snake venoms on a microfluidic blood vessel model that mimics perfused conditions. This was done in an effort to create a model that could predict the effects of these venoms on epithelial barrier function, cell viability and contraction/delamination. The morphological and quantitative data presented in this study show that at least two distinct mechanisms exist by which the endothelial cells are affected by snake venom. The ways by which the endothelial cells are affected by the venoms can be either ‘direct’ (e.g., affect the cells directly, for example, by disrupting the cellular membrane) or indirect (e.g., by affecting the ECM, which could eventually lead to cell injury). Regardless of the mechanisms of endothelial cell damage this may lead to vascular leakage (see Fig. 7)^6,12,28–30^.

The first mechanism includes degradation of the ECM, which is observed as the detachment of the cell monolayer from the surrounding tube. The ECM is made up of the interstitial matrix and the basement membrane, which consist of glycoproteins, proteoglycans and fibrous proteins such as collagen and laminin. The ECM is a macromolecular structure that is important for providing structural support to a variety of cell types^29,31–33^. Because of its pivotal role, the ECM provides an important target for various toxin classes with tissue-damaging activities, including snake venom metalloproteases and hyaluronidases^16,29,34,35^. Degradation of the ECM may affect various cell types, including (skeletal) muscle cells, keratinocytes, kidney cells, and endothelial cells^1,28,29,36–40^. Hydrolysis of the ECM surrounding the capillary endothelial weakens the capillary wall and may ultimately result in vascular leakage^12,13,15,16,29^. This is in line with the observations in this study, in which the detachment of the vessel from its surroundings is accompanied by vascular leakage, which can be seen as the endothelial cells delaminate, as is the case for *E. ocellatus*. In this process, the cell membrane remains intact, shown by the fact that PI is not entering the cell, nor is Calcein-AM leaking out of the cell. The observed effect can, therefore, be considered an indirect cytotoxic effect, which means that cell death occurs as a secondary effect resulting from the ECM degradation and not by directly destroying the endothelial cells^6,12,15^. Initially, proteolytic enzymes degrade the basement membrane and adhesion proteins, thus weakening the capillary wall and perturbing the interactions between endothelial cells and the basement membrane. The pathological effects described in these cells in vivo are therefore argued to be a result of the mechanical action of haemodynamic forces secondarily to the weakening of the basement membrane^12,13,15,29,41,42^. The venoms of *N. mossambica* and *N. naja* did affect the integrity of the vessel to some extent (i.e., lesions can be observed in the top section of the vessel wall and extensive loss of Calcein-AM), the venoms did not seem to affect the ECM of the endothelial cells.

The second mechanism likely involves disruption of the cellular membrane, which can be deduced from the fact that the cell membrane becomes permeable to both PI and Calcein-AM. The destabilisation of the cell membrane is associated with a number of snake venom toxins, including cytotoxic phospholipases A_2_s, cytotoxic three-finger toxins and ß-defensin-like toxins^43,44^. Cellular membranes obviously play a crucial role in normal cell functioning and, therefore, are an optimal target for a variety of toxin classes. These ‘true’ cytotoxins deploy their effects by altering membrane integrity, in particular, the plasma membrane^45^. Disruption of the membrane may occur via hydrolysis of membrane phospholipids, direct perturbation of the membrane, pore-formation and other cell-penetrating mechanisms^43,46–54^. As the venoms of *N. mossambica* and *N. naja* indeed affect cell viability in combination with Calcein-AM leakage from the cells, we can conclude that these venoms have a direct cytotoxic effect. This is in line with observations from previous studies^28^. As cell death occurs preferentially in cells adjacent to apoptotic cells, there might be a mechanism in play that propagates cell death. A possible explanation might be ATP-release secondary to toxin-induced cytotoxicity, which may lead to cell death pathways of neighbouring cells^55–58^.

However, future studies on isolated toxins should focus on the exact mechanisms by which these venoms execute their damaging effects on cells. The venoms of *E. ocellatus* and *B. multicinctus* did not seem to have much impact on membrane stability, as PI uptake and Calcein-AM leakage were similar to negative control. However, the delamination of endothelial tubes for *E. ocellatus* made assessment of cell viability impossible.

## Concluding remarks

In this study, we presented a method to investigate the impact of snake venom on in vitro-grown perfused endothelial tubules that are in direct contact with an ECM. This allows us to study the molecular and cellular mechanisms underlying venom-induced tissue damage in a way that closely resembles conditions in the human body. The use of a microfluidic device replicating the complex physiological micro-environment of the capillary vessel allows for an accurate, sensitive and real-time study of the tissue-damaging effects of snake venoms on barrier integrity. We studied the impact of four medically relevant snake venoms using this platform and identified two mechanisms by which these venoms affected the microvasculature. The ‘direct’ cytotoxic effects of *N.mossambica* and *N. naja* venoms affect the cells by disrupting the cellular membrane, whereas the ‘indirect’ effects of *E. ocellatus* venom affect the ECM, leading to delamination and contraction or collapse of the blood vessel. Based on the findings in this study, the cellular effects of *B. multicinctus* seem to be minimal.

The organ-on-chip approach could provide valuable insights into the pathogenesis of haemorrhagic activities of snake venoms and could be used to assess the neutralising capabilities of snakebite treatments in real-time and in near-native conditions. The approach will thus contribute to the development of better, more effective strategies to mitigate snakebite morbidity while reducing the number of survival studies needed on mice.

### Supplementary Information

Supplementary Information 1.

Supplementary Video 1.

## Data Availability

The datasets used and/or analysed during the current study available from the corresponding author on reasonable request.
