# Author Correction: Using organ-on-a-chip technology to study haemorrhagic activities of snake venoms on endothelial tubules

**DOI:** 10.1038/s41598-024-66606-9

**Published:** 2024-07-08

**Authors:** Mátyás A. Bittenbinder, Flavio Bonanini, Dorota Kurek, Paul Vulto, Jeroen Kool, Freek J. Vonk

**Affiliations:** 1 Naturalis Biodiversity Center, 2333 CR Leiden, The Netherlands; 2 AIMMS, Division of BioAnalytical Chemistry, Department of Chemistry and Pharmaceutical Sciences, Faculty of Sciences, Vrije Universiteit Amsterdam, De Boelelaan 1085, 1081HV Amsterdam, The Netherlands; 3 Mimetas, Leiden, The Netherlands

Correction to: Scientific Reports, 10.1038/s41598-024-60282-5, published online 4 June 2024

The original version of this Article contained an error in Figure 6, where the pictures of the cells in the top row (EcOc) and the bottom row (NaNa) were duplicated. The original Figure 6 and accompanying legend appear below.

**Figure 6:**
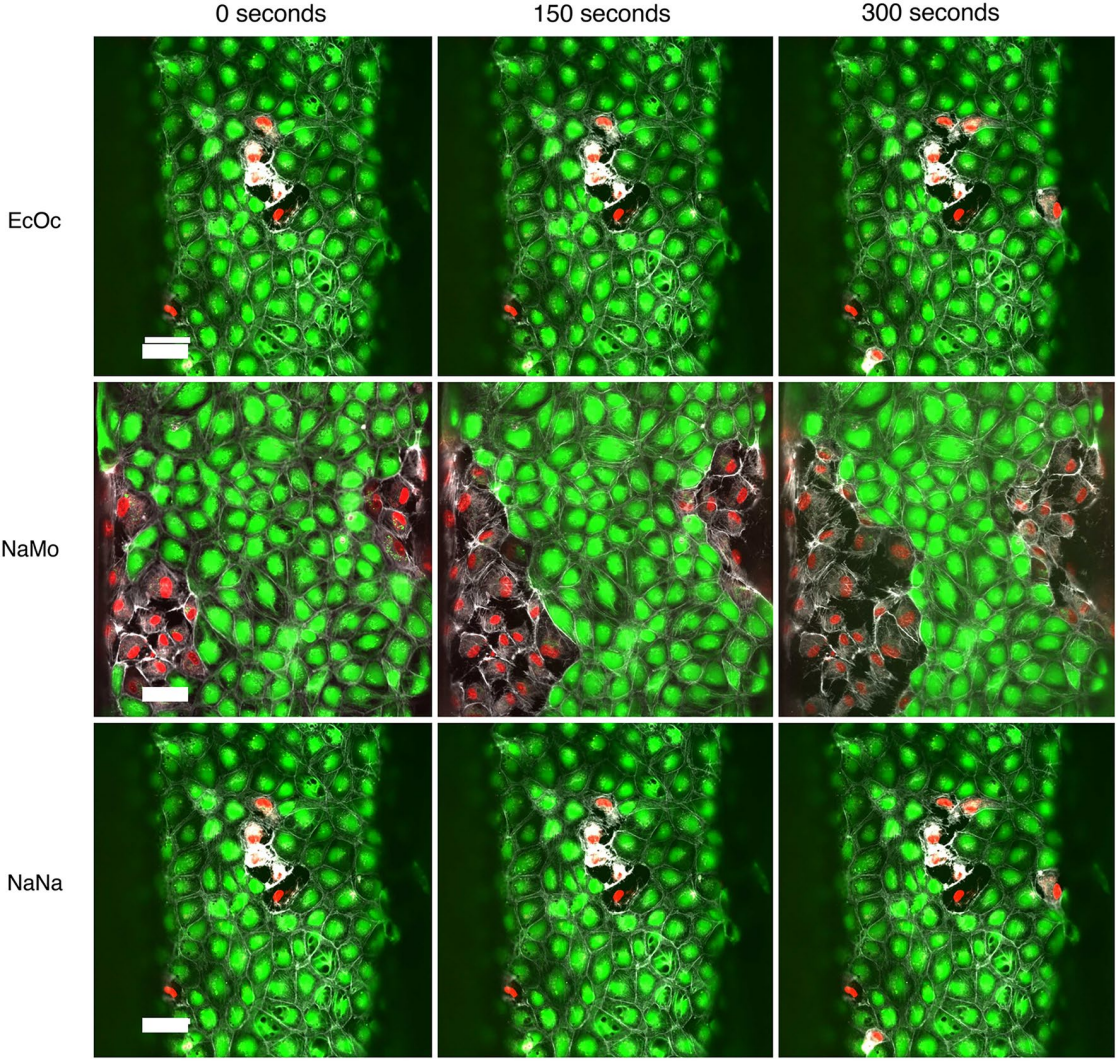
Timelapse of high venom dose exposure on endothelial tubules Immunofluorescent microscopy images show the difference in morphology of the endothelial vessels after 0, 150, and 300 s of exposure to 100 μg/mL of snake venom compared to the control. PI is shown in red, Calcein-AM is shown in green, and live-actin is shown in white. The scale bar represents 50 μm.

The original Article has been corrected.

