# Supplementary material for: Using organ-on-a-chip technology to study haemorrhagic activities of snake venoms on endothelial tubules
Source: Sci Rep. 2024 Jun 4;14:11157. doi: 10.1038/s41598-024-60282-5 (PMC11150252; doi:10.1038/s41598-024-60282-5)
Supplement: Supplementary file 1 — Supplementary Information 1. [file 41598_2024_60282_MOESM1_ESM.docx]

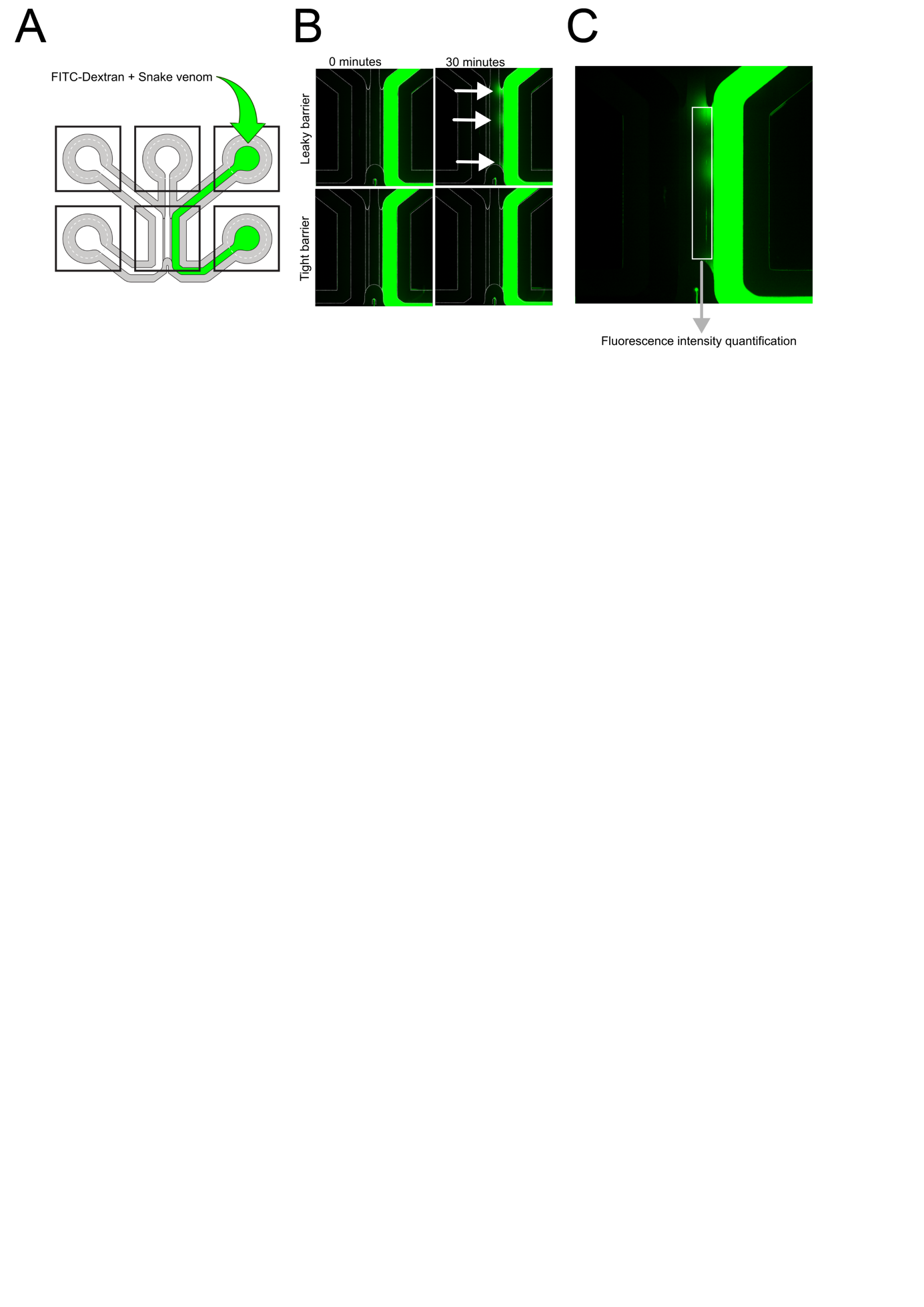


**Supplementary Figure 1**: Vascular leakage assay. (A) FITC-labelled dextran and snake venom are added to the lumen of the blood vessel. (B) Fluorescence microscopy is used to assess the blood vessel barrier integrity. Leaky vessels permit the diffusion of dextran into the adjacent gel channel over time (white arrows indicate leakage). (C) Leakage is quantified by measuring the mean fluorescence intensity in a defined region adjacent to the blood vessel in the gel channel.

**S1 Movie.** Live confocal imaging of the structural collapse of a HUVEC tubule exposed to 100 μg/ml *E.ocellatus* venom. White indicates the actin cytoskeleton, green represents live cells, and red indicates the nuclei of dead cells. The total time elapsed is 370 second, with an image acquired every 10 seconds.
